# Contralateral Hypertrophy Post Yttrium-90 Transarterial Radioembolization in Patients With Hepatocellular Carcinoma and Portal Vein Tumor Thrombus

**DOI:** 10.7759/cureus.59260

**Published:** 2024-04-29

**Authors:** Anastasia Hadjivassiliou, Xinchi Hou, Leandro Cardarelli-Leite, Ivan S Klyuzhin, François Bénard, Darren Klass, Stephen G.F. Ho, Arman Rahmim, David Liu

**Affiliations:** 1 Department of Radiology, University of British Columbia, Vancouver, CAN; 2 Department of Functional Imaging, BC Cancer Research Institute, Vancouver, CAN; 3 Department of Medical Imaging, Western University, London, CAN; 4 Department of Integrative Oncology, BC Cancer Research Institute, Vancouver, CAN; 5 Department of Molecular Oncology, BC Cancer Research Institute, Vancouver, CAN; 6 Department of Functional Imaging, BC Cancer, Vancouver, CAN

**Keywords:** statistical analysis, hepatocellular carcinoma, contralateral hypertrophy, transarterial radioembolization, yttrium-90

## Abstract

Objectives

Contralateral hypertrophy of non-irradiated liver following Yttrium-90 (^90^Y) transarterial radioembolization (TARE) is increasingly recognized as an option to facilitate curative surgical resection in patients that would otherwise not be surgical candidates due to a small future liver remnant (FLR). This study aimed to investigate the correlation between patient features and liver hypertrophy and identify potential predictors for liver growth in patients with hepatocellular carcinoma (HCC) and portal vein tumor thrombus (PVTT) undergoing TARE.

Methodology

Twenty-three patients with HCC and PVTT were included. Contralateral liver hypertrophy was assessed at six months posttreatment based on CT or MRI imaging. Thirteen patient features were selected for statistical and prediction analysis. Univariate Spearman correlation and analysis of variance (ANOVA) tests were performed. Subsequently, four feature-selection methods based on multivariate analysis were used to improve model generalization performance. The selected features were applied to train linear regression models, with fivefold cross-validation to assess the performance of the predicted models.

Results

The ratio of disease-free target liver volume to spared liver volume and total liver volume showed the highest correlations with contralateral hypertrophy (*P*-values = 0.03 and 0.05, respectively). In three out of four feature-selection methods, the feature of disease-free target liver volume to total liver volume ratio was selected, having positive correlations with the outcome and suggesting that more hypertrophy may be expected when more volume of disease-free liver is irradiated.

Conclusions

Contralateral hypertrophy post-^90^Y TARE can be an option for facilitating surgical resection in patients with otherwise small FLR.

## Introduction

Yttrium-90 (^90^Y) transarterial radioembolization (TARE) is a treatment option in selected patients with primary and secondary liver malignancy [1]. Its role in the management of hepatocellular carcinoma (HCC) has been well documented in the literature, with more experience being gained in cholangiocarcinoma in addition to metastatic lesions such as from neuroendocrine, colorectal, or breast malignancies [2]. Initial experience of contralateral hypertrophy in HCC patients post-^90^Y TARE was observed in 2009 [3].

Multiple studies have shown that the non-irradiated liver's contralateral hypertrophy (CH) occurs following TARE [3-6]. This effect has increasingly gained attention as an option for inducing hypertrophy because ^90^Y TARE provides tumoral control while enabling contralateral hypertrophy. This could then facilitate curative surgical resection in patients who would otherwise not be surgical candidates due to a small future liver remnant (FLR) [6-7]. Other treatment strategies utilized to induce liver hypertrophy, such as portal vein embolization (PVE), or associating liver partition and portal vein ligation for staged hepatectomy (ALPPS), have limitations. PVE, for instance, is limited by tumoral progression that occurs, while ALPPS has shown high perioperative morbidity and mortality [7].

The studies published to date regarding post-^90^Y TARE hypertrophy are heterogeneous, which makes extrapolating results for different patient groups challenging. The function of background liver parenchyma, type of tumor, previous treatments, and tumoral morphology (hypovascular versus hypervascular lesions) are important factors that could affect the degree of hypertrophy achieved; however, they vary across studies and are not consistently defined [2]. Identification of such predictors could potentially help guide patient selection or adjustment of dosimetry calculations for optimization of hypertrophy.

In this study, we aim to identify factors from patient demographics, biochemical values, pre-procedural imaging findings, or dosimetry parameters that may predict the degree of contralateral hypertrophy post-^90^Y TARE in patients with HCC and portal vein tumor thrombus (PVTT) that did not receive any prior systemic or locoregional therapies.

## Materials and methods

A retrospective analysis was conducted at a single Canadian tertiary referral center of patients receiving ^90^Y TARE with PVTT between April 2009 and April 2019. The patients had no previous systemic or locoregional treatment. Pre-procedural imaging at baseline (CT or MRI) and follow-up at six months was used for volumetric analysis. Institutional review board (IRB) approval was obtained for data collection and analysis.

Patient characteristics

Twenty-three patients were included in this study. All patients had preserved liver function (Child-Pugh ≤ B7). None of the patients had extrahepatic disease.

Primary outcome

The primary outcome was defined as the percentage growth of spared (nonirradiated) liver in patients, which was calculated using the following formula:

$\frac{Spared \ liver\ volume\ at\ 6\ months-Spared \ liver \ volume \ at \ baseline}{Spared \ liver \ volume \ at \ baseline} \times 100\%$

Statistical analysis

Thirteen input features were considered, as illustrated in Table 1. Biochemistry and lab values were obtained from online medical records as the most recent available preceding the ^90^Y mapping procedure (departmental protocol was within one month). The degree of PVTT involvement was classified according to the Liver Cancer Study Group of Japan. Volumetric analysis and contouring were performed with commercially available software (Osirix) with dosimetric analysis utilizing three-compartment partition modeling (REF) via the Dosimetry and Activity Visualizer for ^90^Y (DAVYR). Three of the patient features were calculated from volumetric measurements using the baseline imaging before TARE (fraction of target liver volume/total liver volume, fraction of disease-free target liver volume/spared liver volume, and fraction of disease-free target liver volume/total liver volume). A summary of these features is listed in Table 1.

**Table 1:** Patient demographics and input features employed in this study.

Patient	*n *= 23
Categorical features, *n* (%)	
Gender	
Female	3 (13%)
Male	20 (77%)
PVTT classification	
Vp1	4 (17%)
Vp2	7 (30%)
Vp3	9 (39%)
Vp4	3 (13%)
Radioembolization product types:	
SIR-Spheres (Resin)	7 (30%)
TheraSphere (Glass)	16 (70%)
Presence of Hepatitis B	6 (26%)
Presence of Hepatitis C	13 (57%)
Presence of ascites at baseline imaging	3 (13%)
Continuous features, median (range)	
Ages at the time of diagnosis (years)	62 (48-75)
Liver dose (Gy)	94.4 (21.0-219.0)
Spleen size (cm)	12.2 (7.2-19.3)
Platelets count (×10^6^/L)	116 (45-368)
Fraction of target liver/total liver at baseline	0.49 (0.13-0.79)
Fraction of disease-free target liver/spared liver at baseline	0.69 (0.08-3.35)
Fraction of disease-free target liver/total liver at baseline	0.37 (0.06-0.72)
Percentage growth of spared liver (%)	22.7 (-33.09 to 147.39)

Univariate analysis was performed to investigate the relationship between the 13 input features and the primary outcome. Spearman correlations test was used for continuous features, and ANOVA test was used for categorical features. Correction for multiple testing was performed using the false discovery rate (FDR) Benjamini-Hochberg (BH) step-up procedure.

Subsequently, multivariate analysis was performed to build models for predicting the primary outcome. To improve model generalization performance and avoid overfitting, feature selection was first performed based on four different methods. Method 1 was based on Spearman and ANOVA tests between input features and outcome (*P*-value < 0.1 as inclusion criterion), followed by correlation measurements between the features. Pairs of all features with Spearman correlations greater than 0.8 were considered redundant and reduced. Method 2 employed the *F*-test to examine the importance of each feature individually, ranking features using *P*-values (*P*-values < 0.1 as inclusion criterion). Methods 3 and 4 were embedded feature selection methods, employing lasso regression and stepwise linear regression with fivefold cross-validation, respectively. Since the selected predictors (features) could be different for each regression run, 100 runs of Methods 3 and 4 were performed. The top five features with the highest selecting frequencies from those 100 runs were considered. The selected features from the above four methods were then applied to train regression models, including linear regressions, regression trees, Gaussian process regression, support vector machines (SVMs), and ensembles of trees using Regression Learner Application (Matlab® 2020b) with fivefold cross-validation. The performances of predicted models were assessed and compared for the feature combinations from the four different feature selection methods.

To investigate potential connections between input features with the increase/decrease of FLR, binary outcome (BO) was also considered in this study. When the spared liver volume decreased (i.e., the percentage growth of spared, non-irradiated liver in patients was <0%), BO was set as 0, while BO was set as 1 when the percentage growth of spared, non-irradiated liver in patients was ≥0%. Feature importance was ranked based on the BO by performing both the chi-square test and the minimum redundancy maximum relevance (MRMR) algorithm.

## Results

Primary outcome

The median percentage growth of the spared liver (primary outcome) was 22.7% (range 33%-147%). Five out of 23 patients demonstrated a decrease in the size of the spared liver at six months.

Patient features

**Figure 1:**
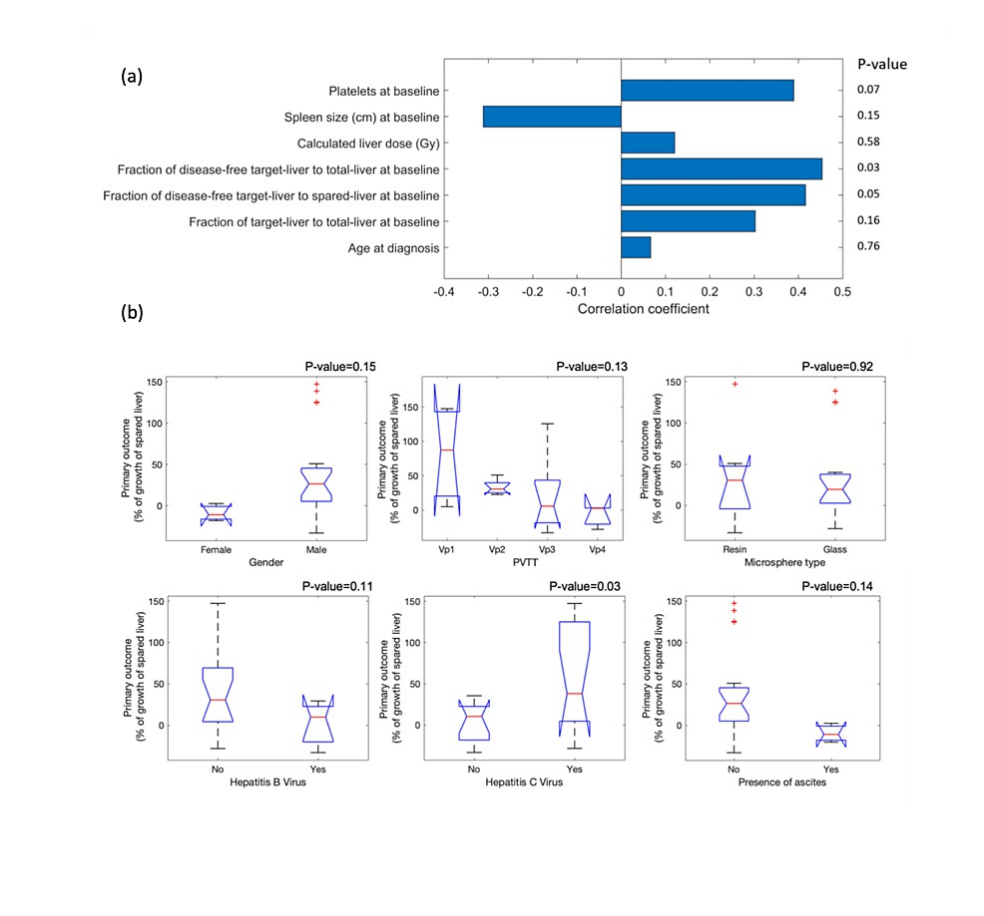
Univariate analysis between 13 input features and primary outcome: (a) Spearman correlation test results for seven continuous features and (b) analysis of variance (ANOVA) test for six categorical features.

All features listed in Table 1 were included in the analysis. Figure 1 depicts relations between the 13 input features and the primary outcome based on Spearman and ANOVA tests (Figures 1a-1b, respectively). Positive correlation coefficients indicate the corresponding feature contributes to the growth of the spared liver. The fraction of disease-free target-liver to total-liver and to spared-liver at baseline, as well as hepatitis C virus status, exhibited the highest correlations (with *P*-values of 0.03, 0.05, and 0.03, respectively). However, after adjustment for false discovery rate (FDR), the univariate significance was not retained.

**Figure 2:**
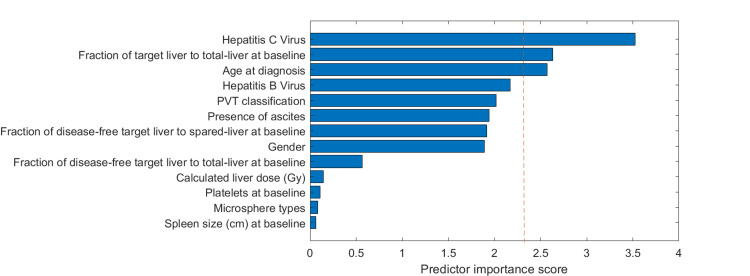
Univariate feature ranking for regression using F-tests. The red dotted line corresponds to a *P*-value of 0.1. The higher scores indicate the most important predictors.

**Figure 3:**
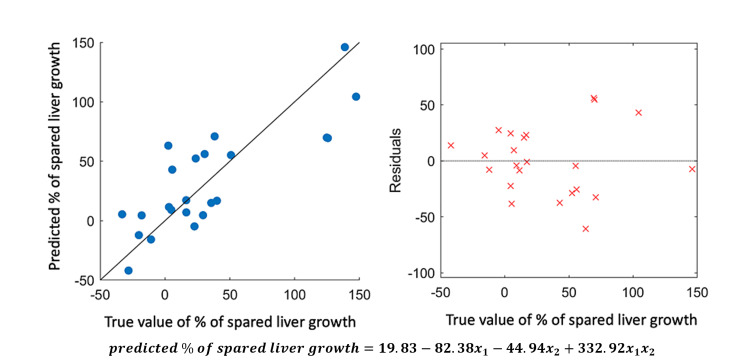
An example of the prediction model from linear regression based on two prominent features. Features x1 and x2 in the formula represent *Hepatitis C Virus* and *Fraction of disease-free target liver to total liver at baseline*, respectively. The validation R-squared performance is 0.60 (fivefold cross-validation). The regression information is listed in Table 3.

**Figure 4:**
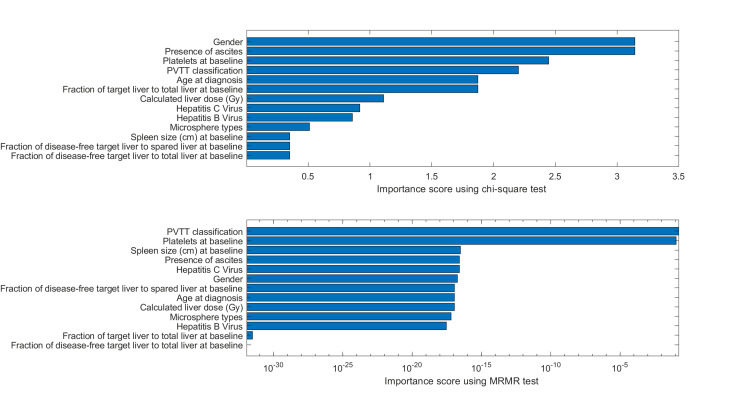
Feature importance ranking with a binary outcome (BO) (BO = 0 when the growth of spared liver <0%, BO = 1 when the growth of spared liver ≥0%) from chi-square tests and MRMR algorithms. MRMR, Minimum Redundancy Maximum Relevance

When using F-tests for feature ranking studies (Method 2), as shown in Figure 2, features of the hepatitis C virus, the fraction of target liver to the total liver at baseline, and patient age were recognized as the most important predictors, with *P*-value < 0.1. Combining with feature selection results from Methods 3 and 4, Table 2 summarizes the selected features. In all four methods, the *Hepatitis C Virus* was selected, and in three out of four methods, the feature *Fraction of disease-free target-liver to total-liver at baseline *was selected. Both features were also the most prominent in univariate analysis. An example of a prediction model from linear regression based on these two prominent features is shown in Figure 3 and Table 3. To investigate the factors influencing the decrease/increase of liver size, Figure 4 shows the feature importance ranking with BOof liver growth.

**Table 2:** Summary of selected features and regression prediction models for the different feature selection methods. *For Methods 3 and 4, the features with the highest popularity in 100 regression runs are listed; the number shown in the bracket indicates feature ranking (similar numbers represent similar rankings). PVTT, portal vein tumor thrombus; SVM, support vector machine

Feature selection	Method 1	Method 2	Method 3*	Method 4*
Selected features	Hepatitis C virus	Hepatitis C virus	Hepatitis C virus (2)	Hepatitis C virus (1)
	Fraction of disease-free target liver to total liver at baseline	Fraction of target liver to total liver at baseline	Fraction of disease-free target liver to total liver at baseline (1)	Fraction of disease-free target liver to total liver at baseline (4)
	Platelets baseline	Age	Platelets baseline (3)	Fraction of disease-free target liver to spared liver at baseline (3)
	-	-	PVTT classification (2)	PVTT classification (2)
	-	-	Presence of ascites baseline (3)	Platelets baseline (5)
Best regression model	SVM	SVM	Linear	Tree
R-squared	0.58	0.51	0.41	0.35

**Table 3:** Linear regression. Data taken from Figure 3. x1 represents hepatitis C virus (HCV) status; x2 represents the fraction of disease-free target liver volume/total liver volume. SE, standard error

Coefficient	Estimate	SE	*t*-Stat	*P*-value
Intercept	19.83	20.41	0.971	0.344
x_1_	-82.38	32	-2.57	0.019
x_2_	-44.94	55.92	-0.8	0.432
x_1_x_2_	332.92	79.9	4.167	5.00E-04

## Discussion

Contralateral hypertrophy post-^90^Y TARE is a recognized phenomenon [3-7]. Liver regeneration and hyperplasia following injury (atrophy-hypertrophy complex), PVE, or hepatic surgical resection is a complex process at the cellular level with multiple pathways involved and is not fully understood [8,9]. It is generally hypothesized that the mechanism of action of ^90^Y TARE is based primarily on radiobiological effects rather than the embolic load of particles [10]. Radiation-induced fibrosis and subsequent atrophy in the part of the liver treated with^ 90^Y is thought to cause slow diversion of portal flow over time to the contralateral lobe thereby increasing its volume.

The satisfactory volume of FLR depends on the background liver function. For patients with normal liver function, an FLR of more than 20% may be suitable, whereas in patients with established cirrhosis, more than 40% is recommended [11,12]. Induction of hepatic hypertrophy to facilitate surgical resection in patients with liver malignancy and inadequate FLR has traditionally been performed with PVE [13]. Other methods, including combined transarterial embolization with PVE, liver venous deprivation, and ALPPS have been used [12]. 

The potential role of ^90^Y TARE as a primary indication to facilitate curative resection in patients with HCC has led to new discussions for the management of patients that would otherwise not be considered surgical candidates due to an inadequate FLR while providing treatment of the liver malignancy [11,14]. Treatment of the liver malignancy is not feasible at the time of PVE or ALPPS. A previous review of studies performed assessing post-^90^Y TARE contralateral hypertrophy showed variation with regards to the degree of hypertrophy that occurs, ranging on average between 26% and 47% over 44 days to nine months [2]. This is thought to be due to heterogeneity across studies regarding patient selection, TARE device used, dosing and administration technique, as well as the timing of hypertrophy assessment.

In the early post-^90^Y TARE period the hypertrophy is slower, with a percentage growth of the FLR of up to 35% seen at three to six months and 45% at nine months [15]. This, however, has been inconsistent as another study showed 45% hypertrophy at 26 weeks [5]. This is significant with regards to the timing of assessment of hypertrophy post-^90^Y TARE and emphasizes the importance of how patient variables can affect the degree of hypertrophy that can occur. It has been suggested that assessment of post-^90^Y TARE hypertrophy should not occur before three months, and if the FLR is not satisfactory, then the assessment should be repeated at six months [16]. PVE induces hypertrophy at a faster rate, on average at six weeks. In a study performed comparing hypertrophy post-^90^Y TARE and PVE, it was found that PVE resulted in more significant liver hypertrophy in a shorter timeframe (61.5%, median 33 days) versus TARE (29%, median 46 days). However, tumoral growth was not assessed [17]. The interval period of achieving hypertrophy is deemed less of a concern with ^90^Y rather than with PVE as the liver malignancy is treated concomitantly. Furthermore, TARE provides an advantage of a biological test of time. If a patient develops new or worsening disease in the irradiated segment or new disease in the contralateral untreated FLR, then this indicates poor tumoral biology and brings into question whether surgical resection would prolong the patient’s overall survival.

Multiple studies have been performed to identify patient factors that may affect the degree of contralateral hypertrophy; however, the results have been inconsistent. This study did not show a significant correlation with regards to indicators of background liver function as seen in prior studies such as the presence of ascites or Child-Pugh Score [16,18,19]. Similarly, a prospective study performed did not identify any predictive factors for post-^90^Y TARE hypertrophy [20]. The results from the feature importance tests showed that platelet count and PVTT classification may be relevant. Patient spleen size may influence the results of decrease/increase of FLR since the platelet count was significantly correlated with patient spleen size (Spearman correlation coefficient was -0.80 and *P*-value < 0.005 after FDR). This is in agreement with other publications, which showed that splenomegaly is negatively correlated with hypertrophy [19]. The presence of PVTT has been described as an *idiopathic *redirection of blood flow to the contralateral liver, and this was found to be positively correlated with hypertrophy in this dataset. Twenty-two percent of patients, however, demonstrated a decrease in the size of the FLR following TARE, which is similar to other publications [20]. The reason for this observation is unclear; it is suspected to reflect a worsening of the background liver disease as well as a decrease in tumor size.

An unexpected finding in these data was the correlation between the percentage of spared liver growth and hepatitis C. Patients with hepatitis B are generally deemed to have more hepatic reserve and can undergo more liver regeneration. A previous study showed that patients with hepatitis B demonstrate more hypertrophy post-^90^Y TARE in comparison to patients with hepatitis C or alcoholic liver disease [18]. It is uncertain whether the result in this study was due to the small number of patients with background hepatitis B in this sample.

In the surgical literature, more percentage growth of remnant liver is seen following extensive resections. This suggests that the larger the volume of functional liver that is removed, the more regeneration needs to occur for compensation. Extrapolating this to ^90^Y TARE raises the question of whether a more proximal ‘whole lobe’ administration to facilitate atrophy of a larger part of the liver should be performed (provided that the patient can tolerate this), regardless of whether the tumor could be treated with a smaller, targeted administration. The data from this study support that the more the non-tumor-bearing liver is irradiated, the more contralateral hypertrophy is seen. This is the first study where this observation has been made. It is assumed that liver parenchyma affected by tumor is not deemed to have the same function as parenchyma that is disease-free; therefore, in patients where the irradiated segment consists of almost entirely tumor, minimal normal liver parenchyma would be sacrificed; the requirement for hypertrophy to compensate would, therefore, be expected to be low. 

This study’s findings provide two important considerations with regard to dosimetry. The first is that where a tumor can be anatomically treated with only a small liver area being irradiated, one should consider whether a larger volume of the liver should be treated instead. The technique of having two administrations (one with a high dose to treat the tumor, with a second *booster* administration of a lower dose to non-tumoral liver parenchyma) has been described previously [21, 22]. In this institutional experience, a single administration is safe [23]. The second consideration is when performing TARE on lesions that have replaced most of the liver parenchyma. If contralateral hypertrophy would be one of the treatment intents, contemplating systemic chemotherapy first to decrease the tumoral size (and therefore restore more normal parenchyma in the area to be targeted with radiation) could be an option before performing TARE, bearing in mind that exposure of the liver to chemotherapy may affect the ability to regenerate.

The question of optimizing or personalizing dosimetry to achieve hypertrophy is an important topic to explore. This study did not demonstrate a statistically significant correlation between dose to the liver parenchyma and the percentage of liver growth which is in line with previous findings [4]. The possibility of a threshold dose that should be delivered to the liver parenchyma has been described, with a suggestion that if the liver received 88 mGy, >10% of maximal liver hypertrophy was seen [16]. In this study, the median dose for patients treated with TheraSphere was 115.5 Gy and with SIR-Spheres 74.5 Gy. This raises the possibility that the lack of demonstrating a statistically significant relationship between parenchymal dose and degree of hypertrophy in this data was because the dose utilized was overall high and may have exceeded a *threshold*. It remains to be answered how to extrapolate the dosimetry data between TheraSphere and SIR-Spheres, given that the two products are considered to be different. There was no difference found in the hypertrophy induced between the two radioembolization products in this study. However, the number of patients included is too small and unlikely to detect small statistically significant changes.

The point of administration (proximal versus selective), dosimetric calculations (dose to tumor, dose to normal liver parenchyma), and device to be used (considering distribution and particle load) are variables that could be actively changed by the operator and lead to a change in practice. Patient-specific characteristics are required for stratification purposes to identify which patients would achieve hypertrophy and thus benefit from 90Y TARE, as opposed to patients who would not undergo regeneration and, therefore, might undergo TARE unnecessarily. It is difficult to know whether the patients who would not benefit from ^90^Y TARE for hypertrophy would respond differently if they underwent alternative methods such as PVE instead.

This study is limited by the small sample size and retrospective nature, which makes it difficult to identify statistical significance with variables and contralateral hypertrophy. Strict exclusion criteria were applied to provide a homogeneous patient cohort with no prior treatments. The disadvantage is that this limits the ability to detect small statistically significant changes. A larger sample size would also potentially allow the use of radiomics [24-26] and dosiomics frameworks to correlate patient image and dose distribution to outcomes for improving predictive modeling [27-29].

## Conclusions

More knowledge has been gained with 90Y TARE, leading to an overall shift toward personalized dosimetry aimed at improving patient outcomes. This study included statistical analyses and prediction models of contralateral liver hypertrophy in patients with HCC and PVTT post-^90^Y TARE. The findings raise considerations for patient-specific HCC treatment, optimization of patient stratification, and dosimetric calculations to enable patients with small FLR to achieve curative resection.
